# Breath Pentane as a Potential Biomarker for Survival in Hepatic Ischemia and Reperfusion Injury—A Pilot Study

**DOI:** 10.1371/journal.pone.0044940

**Published:** 2012-09-11

**Authors:** Changsong Wang, Jinghui Shi, Bo Sun, Desheng Liu, Peng Li, Yulei Gong, Ying He, Shujuan Liu, Guowang Xu, Jianyi Li, Ailin Luo, Enyou Li

**Affiliations:** 1 Department of Anesthesiology, The First Affiliated Hospital of Harbin Medical University, Harbin, China; 2 Department of Anesthesiology, Tongji Hospital of Tongji Medical College, Huazhong University of Science and Technology, Wuhan, China; 3 CAS Key Laboratory of Separation Science for Analytical Chemistry, Dalian Institute of Chemical Physics, Chinese Academy of Sciences, Dalian, China; 4 The Third High School of Harbin, Harbin, China; Institute of Hepatology London, United Kingdom

## Abstract

**Background:**

Exhaled pentane, which is produced as a consequence of reactive oxygen species-mediated lipid peroxidation, is a marker of oxidative stress. Propofol is widely used as a hypnotic agent in intensive care units and the operating room. Moreover, this agent has been reported to inhibit lipid peroxidation by directly scavenging reactive oxygen species. In this study, using a porcine liver ischemia-reperfusion injury model, we have evaluated the hypothesis that high concentrations of breath pentane are related to adverse outcome and that propofol could reduce breath pentane and improve liver injury and outcome in swine in this situation.

**Methodology/Principal Findings:**

Twenty male swine were assigned to two groups: propofol (n = 10) and chloral hydrate groups (n = 10). Hepatic ischemia was induced by occluding the portal inflow vessels. Ischemia lasted for 30 min, followed by reperfusion for 360 min. Exhaled and blood pentane concentrations in the chloral hydrate group markedly increased 1 min after reperfusion and then decreased to baseline. Breath and blood pentane concentrations in the propofol group increased 1 min after reperfusion but were significantly lower than in the chloral hydrate group. A negative correlation was found between breath pentane levels and survival in the chloral hydrate group. The median overall survival was 251 min after reperfusion (range 150–360 min) in the chloral hydrate group. All of the swine were alive in the propofol group.

**Conclusions:**

Monitoring of exhaled pentane may be useful for evaluating the severity of hepatic ischemia-reperfusion injury and aid in predicting the outcome; propofol may improve the outcome in this situation.

## Introduction

Ischemia-reperfusion (IR) injury resulting from the interruption and restoration of blood flow is related to a rapid increase in free radical-mediated lipid oxidation and the disruption of cell membrane integrity that can ultimately result in cell death and serious pathophysiological consequences [1]–[3]. Breath pentane, which is produced by reactive oxygen species(ROS) mediated lipid peroxidation of n-6 polyunsaturated membrane fatty acids, is a marker of oxidative stress [4]. In a preliminary study, we demonstrated that breath pentane analysis could provide an early, rapid, noninvasive and continuous assessment of lipid peroxidation during hepatic ischemia–reperfusion injury [5].

Oxidative stress plays a crucial role in many clinical situations, such as sepsis, multiple organ failure and IR injury [1], [6]. As an indicator of lipid peroxidation (an important part of oxidative stress), breath pentane has been shown to be associated with outcome in sick preterm infants [7], [8].

Propofol is a potent intravenous hypnotic drug that is widely used in the operating room for inducing and maintaining anesthesia and in intensive care units for longer-term sedation. As an antioxidant drug, propofol has been reported to inhibit lipid peroxidation and protect cells against oxidative stress in various clinical and animal studies [9]–[11].

Whether breath pentane is associated with liver injury and outcome in a porcine liver IR injury model is unknown; in this study, we evaluated the hypothesis that high concentrations of breath pentane are correlated with adverse outcome using a porcine liver IR injury model. Moreover, propofol anesthesia was set as a control to determine whether propofol could reduce breath pentane and improve liver injury and outcome in swine in this situation.

## Materials and Methods

### Animals

Twenty male swine (3–3.5 months old; 45–55 kg body weight) were involved in this study. All of the swine were acclimatized for at least 7 d before the experiment. The animals were kept on a 12-h light-dark cycle in a temperature-controlled (21°C) room. Twelve hours before the start of the experiment, the animals were fasted with water *ad libitum*. The study protocol was approved by the Animal Care and Use Committee of Harbin Medical University (hmu201106) and conformed to the Guide for the Care and Use of Laboratory Animals [12]. The animals were divided into two groups based on the anesthesia used: propofol (n = 10) and chloral hydrate (n = 10).

### Surgical Preparation

All of the animals were premedicated with 10 mg/kg ketamine intra-muscular (IM), 0.2 mg/kg diazepam IM and 0.05 mg/kg atropine IM. In the propofol group, anesthesia was induced with intravenously 1.5 mg/kg propofol and maintained with 8–10 mg/kg/h propofol, 5 µg/kg fentanyl and 1 mg/kg rocuronium. In the chloral hydrate group, anesthesia was induced with 0.5 g/kg chloral hydrate and maintained with 25–30 g/kg/h chloral hydrate, 5 µg/kg fentanyl and 1 mg/kg rocuronium. The trachea was intubated with a 6.0-mm external diameter cuffed tube. The lungs were mechanically ventilated with an anesthesia machine (Dräger Fabius GS, Germany) with a tidal volume of 10–15 ml/kg, a respiratory rate of 12–15 breaths/min and an inspiration/expiration rate of 1∶2 to maintain P_ET_CO_2_ and PaO_2_ at 30–40 mmHg and over 150 mmHg, respectively, under 30–40% oxygen in nitrogen. Following sterile cervical dissection, the internal jugular vein was cannulated with a 7-Fr double-lumen central venous catheter through which fluids (normal saline at 10 ml/kg/h) and anesthetics were administered. The carotid artery was cannulated with a 20-G arterial catheter to monitor arterial pressure and obtain blood samples. The rectal temperature was also monitored and maintained at 38±1.0°C with a heating blanket. Vasoactive agents, bicarbonate (5%), furosemide, and hydroxyethyl starch 130/0.4 (Voluven) were administered to maintain stable hemodynamics, normal blood pH and urine volume.

### Surgical Procedure

All of the surgical procedures were conducted under aseptic conditions. The swine underwent laparotomy with a Mercedes-type incision, and the inflow of the liver blood was occluded by the Pringle maneuver. A 5-Fr single-lumen central venous catheter was inserted into the inferior vena cava (near the second porta hepatis) for blood sampling. Prior to clamping, 1 mg/kg heparin was iv administered. In both groups, the inflow of liver blood was occluded for 30 min at normothermia, and reperfusion was performed for 360 min. After the experiment, the animals were sacrificed by the iv administration of potassium chloride.

### Experimental Protocol

The whole experiment was divided into four parts. The first part was a 60-min high-flow washout period (fresh gas flow was maintained at 3 L/min, all invasive operations were completed except clamping); the second part was a 60-min stabilization period; the third part was a 30-min ischemia period; and the fourth part was a 360-min reperfusion period (reperfusion for 360 min was the censored point of observation).

### Breath Sampling

Before the experiment, a room air sample (20 ml) was taken. A Teflon T-piece (with septum) was incorporated into the respiratory circuit near the expiratory limb end and used to draw 20-ml breath samples into a gas-tight syringe (50 ml) (Agilent Inc., USA). These samples were transferred immediately into evacuated 20-ml glass vials (Supelco Inc., USA). Breath samples were taken at the following time points: 15, 30, 45 and 60 min during the high-flow washout period; 15, 30, 45 and 60 min during the stabilization period; 1, 15 and 30 min during the ischemia period; and 1, 15, 30, 60, 120 and 180 min during the reperfusion period.

### Blood Sampling

For the measurement of the liver aspartate aminotransferase (AST), malondialdehyde (MDA) and arterial blood gases, arterial blood samples (9 ml) were withdrawn after intubation and 1, 60, 120 and 180 min after reperfusion. The blood samples were centrifuged immediately (3000 rpm, 10 min), and the plasma was separated. The plasma was stored at −80°C until analysis. The AST activity in the plasma was measured using a self-analyzer (Olympus AU5400, Tokyo, Japan). The amount of MDA was measured by chemical chromatometry. Arterial blood gases were measured using a self-analyzer (Roche OPTI CCA, Switzerland). Blood samples from the inferior vena cava (5 ml) were also collected at the following time points: the end of the stabilization period (basal); 1, 15 and 30 min after ischemia; and 1, 15, 30, 60, 120 and 180 min after reperfusion. These blood samples were transferred immediately into evacuated 20-ml glass vials (Supelco Inc., USA) for pentane measurement.

### Pentane Analysis

We have previously described the method of pentane analysis in both breath and blood in detail [5]. Briefly, pentane in breath and blood were preconcentrated by solid-phase microextraction (SPME). SPME devices and 75-µm bonded polydimethylsiloxane/Carboxen-coated fiber assemblies were purchased from Supelco (USA). A gas chromatogram-mass spectrogram (GC-MS, QP 2010, Shimadzu, Japan) equipped with a GS-GasPro (60 m×0.32 mm) plot column (Agilent Technologies, USA) was used for the pentane assay. The external standard method was used to calibrate pentane concentration. Peak areas of 0.5, 1.0, 2.0, 5.0 and 10.0 parts per billion (ppb) of standard pentane were processed by linear regression. A calibration curve was linear, with a correlation coefficient of at least 0.99 over the range of 0.5–10.0 ppb. To assess and ensure the reproducibility and stability of the GC-MS, 2 ppb of pentane was analyzed daily. The within-day and between-day variations were <5% and <10% (relative standard deviation), respectively.

### Histopathological Examination

Liver tissues were collected before ischemia and 90 min after reperfusion from the left and right of the liver, fixed in 10% neutral buffered formalin, embedded in paraffin, sectioned at 5 µm, and stained with hematoxylin and eosin. The samples were observed under a light microscope to examine histopathological changes.

### Statistical Analysis

The sample size was calculated on the basis of previous work in which the survival rate at 360 min following reperfusion was 0.3 in the chloral hydrate group compared with 1.0 in the propofol group by using freedman model. A sample size of n = 19 was computed (the chloral hydrate group : the propofol group = 10∶ 9) with a statistical power of 0.818 to detect a difference with an α error level of 0.05. The statistical analysis was performed using SPSS 13.0 (USA). All data are expressed as the mean±SEM. Intra- and inter-group comparisons were performed by a one-way repeated measures ANOVA, followed by a post-hoc least significant difference test and either an unpaired t-test or Mann-Whitney U test, respectively. The correlation between breath pentane levels and animal survival following hepatic IR injury was examined using Pearson’s correlation coefficients. The Kaplan-Meier method was used to estimate survival time in the two groups. *P*<0.05 was considered significant.

## Results

There were no significant differences in heart rate, mean arterial pressure, central venous pressure, respiratory rate or body temperature between groups (data not shown).

### Breath Pentane

**Figure 1:**
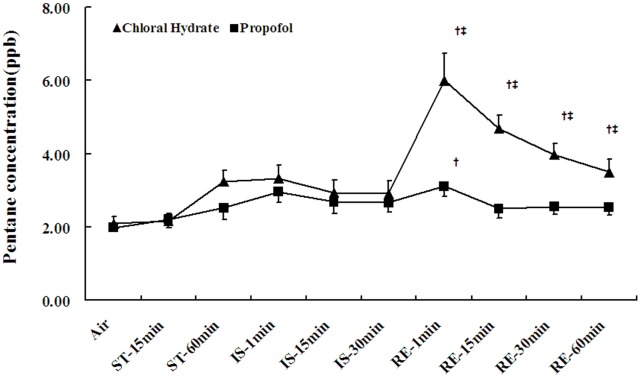
Changes in breath pentane concentrations. The values are expressed as the mean±SE. ST, stabilization period; IS, ischemia period; RE, reperfusion period. A 60-min stretch from the stabilization period was used as the baseline for the breath pentane concentration. ^†^ *P*<0.05 versus the group baseline, ^‡^ *P*<0.05 between groups.

The exhaled pentane concentrations in the chloral hydrate group markedly increased 1 min after reperfusion (P<0.05) and then gradually decreased, whereas the concentrations in the propofol group increased (P<0.05) and then decreased to baseline within 15 min of reperfusion (Figure 1). The peak exhaled pentane concentration of the chloral hydrate group was much higher than that of the propofol group (P<0.05).

### Blood Pentane

**Figure 2:**
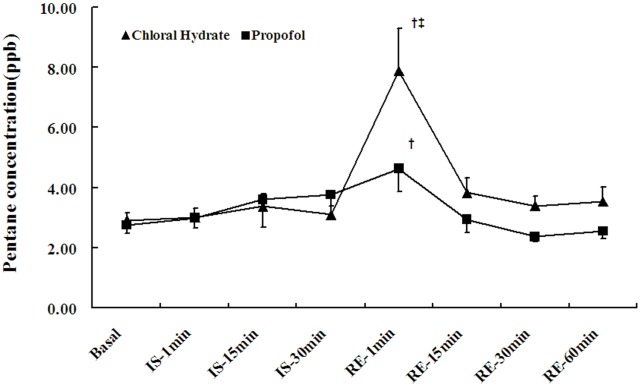
Changes in blood pentane concentrations. The values are expressed as the mean±SE. ST, stabilization period; IS, ischemia period; RE, reperfusion period. A 60-min stretch from the stabilization period was used as the baseline for the breath pentane concentration. ^†^ *P*<0.05 versus the group baseline, ^‡^ *P*<0.05 between groups.

Similar to exhaled pentane, blood pentane concentrations in the chloral hydrate group markedly increased 1 min after reperfusion (P<0.05) but decreased to baseline within 15 min after reperfusion. The concentrations in the propofol group increased 1 min after reperfusion (P<0.05) (Figure 2). The peak blood pentane concentration of the chloral hydrate group was much higher than that of the propofol group (P<0.05).

### Changes in Plasma AST and MDA

**Figure 3:**
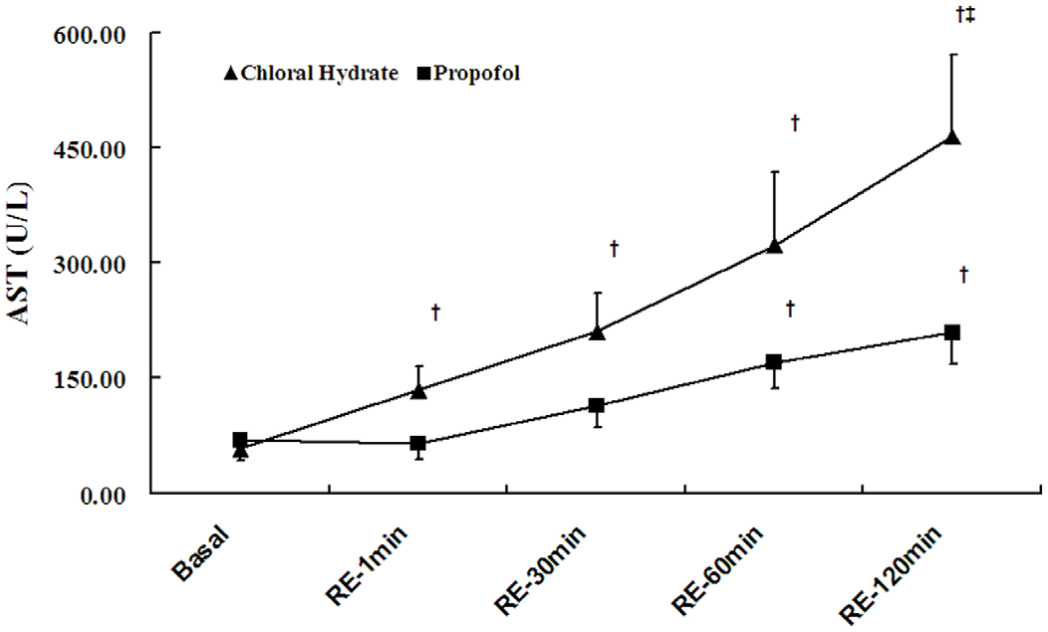
Changes in plasma aspartate aminotransferase (AST). The values are expressed as the mean±SE. RE, reperfusion period. A 15-min stretch from the high-flow washout period was used as a baseline for AST.^ †^ *P*<0.05 versus the group baseline, ^‡^ *P*<0.05 between groups.

**Figure 4:**
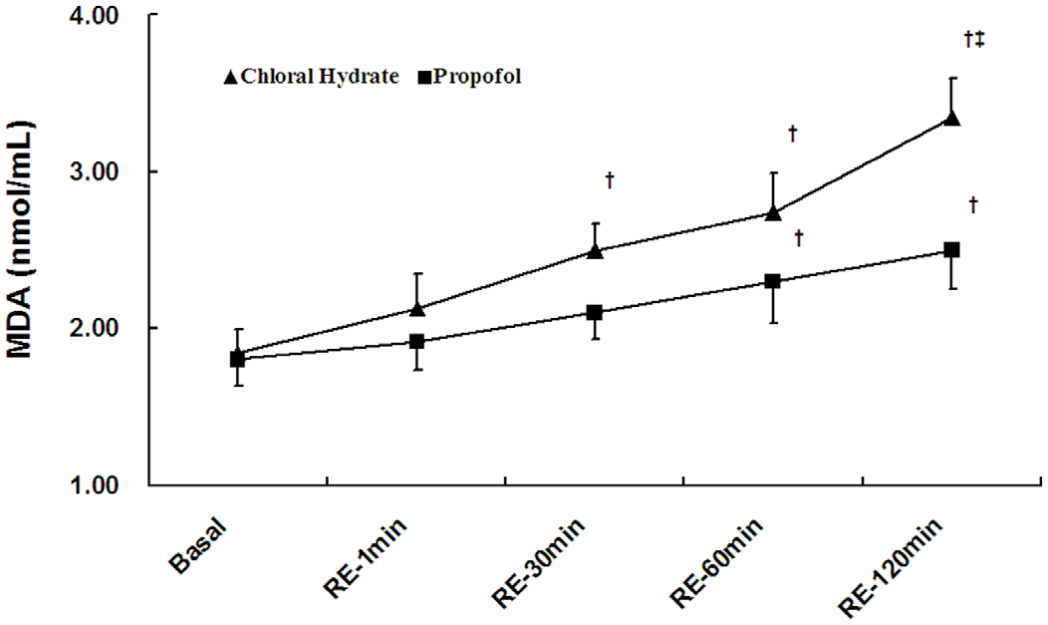
Changes in plasma malondialdehyde (MDA). The values are expressed as the means±SE. RE, reperfusion period. 15 min of high-flow washout period as baseline-MDA.^ †^ *P*<0.05 versus the group baseline, ^‡^ *P*<0.05 between groups.

Plasma AST was elevated in a time-dependent manner after reperfusion in both groups, but the increase in the chloral hydrate group was much greater than in the propofol group (P<0.05, Figure 3). Similar to plasma AST, plasma MDA increased in a time-dependent manner after reperfusion in both groups, but the elevation in the chloral hydrate group was more rapid. At 120 min after reperfusion, the increase in plasma AST in the chloral hydrate group was much greater than in the propofol group (P<0.05, Figure 4).

### Blood Gas Analysis

In the present study, arterial pH was also measured before ischemia and 1 min after hepatic IR. There were no significant differences in PCO_2_, PO_2_, Na^+^, Ca^2+^, tHb, SO_2_% and Hct%. However, significant differences in pH, BE and K^+^ were observed before and after ischemia-reperfusion (Table 1). Moreover, the chloral hydrate group exhibited more severe metabolic acidosis.

**Table 1:** Comparison blood gas analysis in swine before and after hepatic ischemia-reperfusion.

	Chloral hydrate		Propofol	
Parameter	Baseline	After HIR	Baseline	After HIR
pH	7.42±0.02	7.26±0.02*	7.41±0.02	7.35±0.01* ^#^
PCO_2_ (mmHg)	38.80±1.10	40.30±0.92	41.30±0.79	42.70±1.33
PO_2_ (mmHg)	167.80±12.10	134.50±18.38	135.9±17.10	164.90±16.91
BE (mmol/L)	−0.21±0.82	−6.82±1.53*	1.39±0.82	1.45±0.74#
Na^+^ (mmol/L)	145.20±0.65	147.80±0.87	146.70±0.97	146.80±1.05
K^+^ (mmol/L)	3.48±0.09	4.27±0.19*	3.43±0.15	3.83±0.13#
Ca^2+^ (mmol/L)	1.05±0.05	1.00±0.09	0.971±0.03	0.96±0.03
tHb (g/dl)	7.53±0.19	7.39±0.81	6.95±0.31	6.90±0.26
SO_2_%	98.40±0.42	95.20±1.74	94.9±1.87	97.70±0.58
Hct%	21.30±0.62	21.7±0.52	20.7±0.92	20.70±0.80

Values are mean ± SE.

* *P*<0.05 versus the group baseline;

# *P*<0.05 between groups.

After HIR, 1 min after hepatic ischemia-reperfusion.

### Histopathological Examination

**Figure 5:**
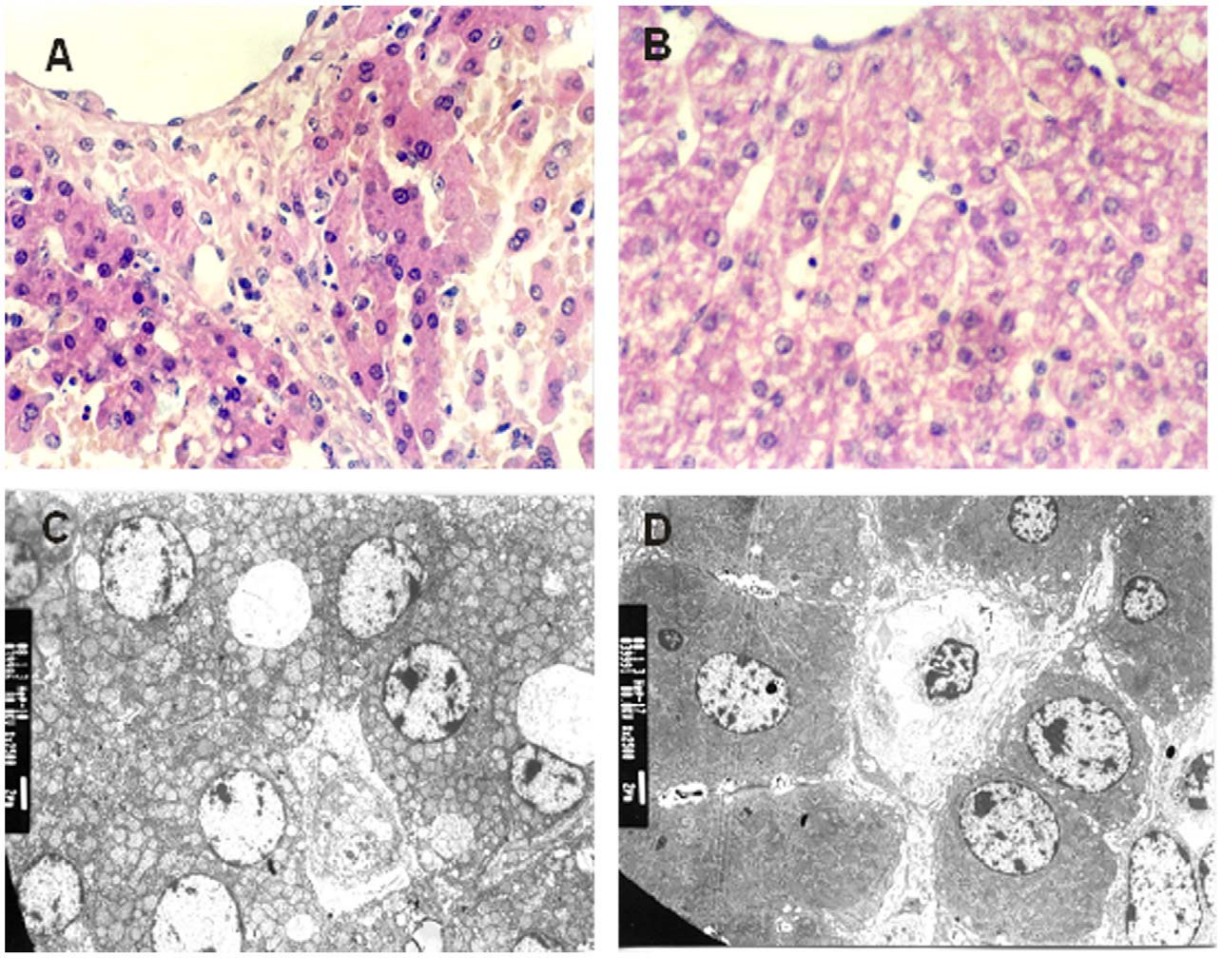
Histopathology of the liver using light (A/B) and electron (C/D) microscopy. A: Congestion, degeneration and necrosis in the centrilobular portion of a liver from the chloral hydrate group (HE×400). B: Inflammatory cell infiltration and the degeneration of granules in the centrilobular portion of a liver from the propofol group (HE×400). C: Accumulation of high density plaques in endonuclear chromatin, bubble degeneration of mitochondria, and the disruption of hepatic sinusoid endothelium in the chloral hydrate group (×1000). D: Complete membrane and a few changes in the nuclear membrane and nucleoli in the propofol group (×1000).

Before ischemia, the histopathological analysis (light microscopy) indicated normal liver structure in both groups. However, in the chloral hydrate group following reperfusion, there was swelling of hepatocytes, congestion, and degeneration in the centrilobular portion of the liver, and dotted necrosis of hepatocytes. In the propofol group, the pathological changes were less pronounced compared with the chloral hydrate group. Electron microscopy also showed swelling, destruction, bubble degeneration of mitochondria, and degranulation of the rough endoplasm in the chloral hydrate group, whereas only mitochondrial swelling was observed in the propofol group (Figure 5).

### Breath Pentane and Survival

**Figure 6:**
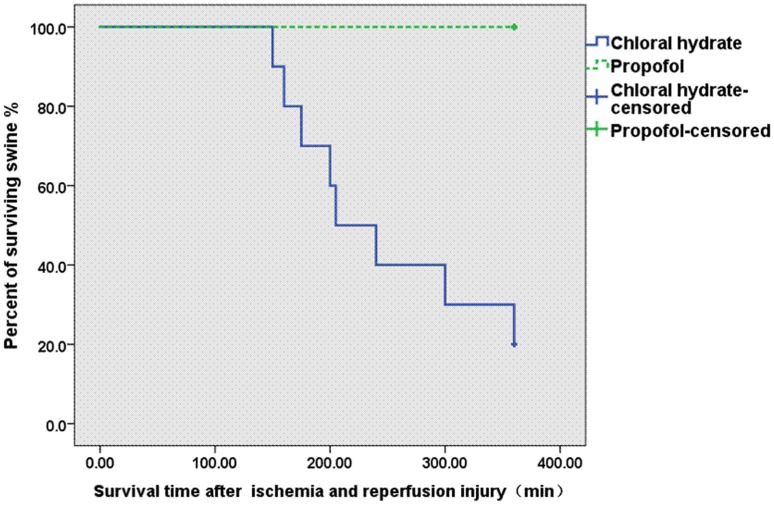
Kaplan-Meier survival plots for the swine in the chloral hydrate (n = 10) and propofol groups (n = 10). Log rank (Mantel-Cox) *P*<0.05.

**Figure 7:**
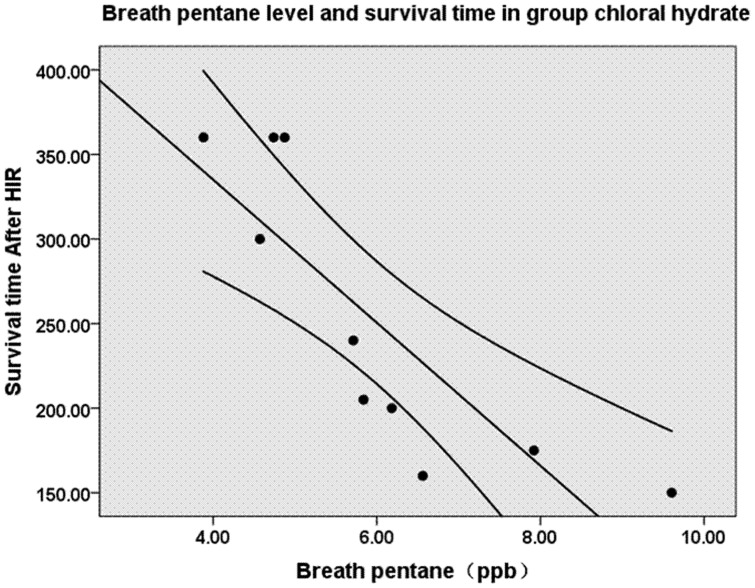
Correlation between breath pentane levels and survival following hepatic ischemia-reperfusion in the chloral hydrate group (n = 10). Regression lines with 95% confidence intervals. HIR, hepatic ischemia-reperfusion. Pearson correlation, −0.840. P<0.05.

The survival rate was 30% at 360 min following reperfusion in the chloral hydrate group, whereas all of the swine in the propofol group survived (Figure 6). The median overall survival duration was 251 min after reperfusion (range 150–360 min) in the chloral hydrate group. Interestingly, there was a significant correlation between breath pentane and survival following hepatic IR injury (r = 0.840, P<0.05, Figure 7).

## Discussion

IR injury triggers a severe oxidative stress that leads to increased lipid peroxidation [13]–[15]. As a chain reaction, lipid peroxidation is initiated by the removal of an allylic hydrogen atom by ROS. The radical generated in this way is conjugated, peroxidized by oxygen, and undergoes several further reactions [16] Aldehydes, such as MDA, are generated along this pathway [17]. Saturated hydrocarbons, such as pentane and ethane, are generated in the same manner from n-6 fatty acids, which are the basic components of cell membranes. Risby *et al.* have produced some works on this topic [18]–[24]. They document that ethane and pentane can simply and quickly reflect the degree of oxidative stress in many clinical situations, such as orthotopic liver transplants, supraceliac aortic crossclamping and cardiopulmonary bypass. In this study, except the relationship between breath pentane and the degree of oxidative stress injury, we also evaluated the hypothesis that high concentrations of breath pentane are correlated with adverse outcome using a porcine liver IR injury model.

In this study, we found that both breath and blood pentane peak 1 min after reperfusion in both groups. Plasma MDA was also significantly increased following reperfusion, but this increase was delayed. Plasma MDA increased later than pentane because when pentane and MDA were generated during lipid peroxidation, both were released into the blood. After circulating in the blood, the pentane diffused across the alveolar–capillary membrane and was then excreted in the breath, but the MDA was diluted by the blood. These results for MDA are due to its accumulation in the blood. Compared with measuring biomarkers in the blood, measuring pentane in breath samples is a non-invasive technique and the sampling does not require skilled medical staff [25]. Moreover, this technique reduces the probability of iatrogenic infection for patients and medical staff because no blood samples are required.

The most prevalent mechanism of cell death induced by extracellular reactive oxygen species is lipid peroxidation. This free radical-mediated process leads to the destruction of cell membranes and can therefore kill hepatocytes very rapidly [26]–[28]. Oxidative stress can also induce apoptosis and necrosis. The mechanism of ROS -induced cell killing involves the promotion of mitochondrial dysfunction through an intracellular oxidant stress in hepatocytes, mainly leading to oncotic necrosis with little apoptosis [26]. The additional release of cell content amplifies the inflammatory injury. In this study, only light and electron micrographs has been done for the histopathological examination. From this result we can only find out in the chloral hydrate group IR caused more severe injury compare with propofol group. We did not perform immunohistochemistry or immunoblotting tests. Therefore, there was not sufficient evidence to conclude that ROS predominantly caused necrosis and very little apoptosis in this situation. This lack of information is one of the limitations of the present study.

Propofol has been reported to modulate IR injury in several organs, suggesting its potential for organ protection during surgery [29]–[32] and possibly as a means to improve patient outcome [33]. Several papers have demonstrated that propofol acts as a scavenger of ROS, decreasing lipid peroxidation in the liver, kidney, heart, and lungs [34], [35].

In this study, oxidative stress was not the direct cause of death. The direct cause of the death was cardiac arrest, not oxidative stress. Cardiac arrest may be due to metabolic acidosis. This phenomenon was similar to that previously reported [36]. IR leads to intensive metabolic acidosis, which, in turn, promotes lipid peroxidation and oxidative stress [37]–[39]; simultaneously, IR leads to intensive oxidative stress, which enhances acidosis [40], [41]. The concentration of pentane represents the final result of lipid peroxidation (lipid peroxidation due to IR and acidosis).

In addition to inhibiting lipid peroxidation, propofol could attenuate hepatic injury caused by IR injury through many other possible mechanisms. In this study, propofol may have reduced inflammatory responses and prevented the development of metabolic acidosis [42], [43]. Moreover, propofol protects hepatic cells from H_2_O_2_-induced apoptosis, partly through activating the MEK-ERK pathway and further suppressing Bad and Bax expression [44]. Propofol ameliorates rat liver IR injury, possibly by inhibiting nuclear factor-kappaB expression [45]. However, further investigation is needed to elucidate the exact mechanism by which propofol attenuates hepatic injury.

Chloral hydrate was used as an agent without known antioxidant properties. Because there were no significant differences in heart rate, mean arterial pressure, central venous pressure, or body temperature between the propofol and chloral hydrate groups, both anesthesia regimens could equally reduce surgical stress, and oxidative stress would not be influenced by surgical stress.

Previous studies showed that exhaled pentane was correlated with the course of neonatal disease and outcome [8]. Crohns et al [46] reported that lung cancer patients with exhaled pentane above the median survived longer than patients with levels below the median. In this study, Kaplan-Meier survival plots clearly indicated that chloral hydrate anesthesia resulted in worse survival (30%) compared with propofol (100%). Breath pentane was also significantly higher in the chloral hydrate group than the propofol group. In addition, there was a negative correlation between breath pentane and survival. Therefore, propofol may improve survival duration with a reduction in pentane production in hepatic IR injury.

The enzyme AST plays a role in the metabolism of alanine. AST is found in high concentrations in liver cells. An increase in AST levels indicates liver damage or disease. In the present study, arterial pH was also measured before ischemia and after reperfusion, and we found that the chloral hydrate group exhibited more severe metabolic acidosis. We acknowledge that if serum lactate, serum INR and serum creatinine were detected in the present study, more useful information on liver function would have been obtained. This lack of information is one of the limitations of the present study.

### Conclusion

Monitoring exhaled pentane may be useful for evaluating the severity of hepatic IR injury and aid in predicting outcome; propofol may improve the outcome in this situation, suggesting its potential benefits when the drug is employed during transplantation surgery and critical illness.
